# Genetic polymorphisms of inflammasome genes associated with pediatric acute lymphoblastic leukemia and clinical prognosis in the Brazilian Amazon

**DOI:** 10.1038/s41598-021-89310-4

**Published:** 2021-05-10

**Authors:** Fabíola Silva Alves, Lilyane Amorim Xabregas, Marlon Wendell Athaydes Kerr, Gláucia Lima Souza, Daniele Sá Pereira, Fábio Magalhães-Gama, Mirian Rodrigues Ribeiro Santiago, Nadja Pinto Garcia, Andréa Monteiro Tarragô, Maurício Morishi Ogusku, Aya Sadahiro, Adriana Malheiro, Allyson Guimarães Costa

**Affiliations:** 1 Programa de Pós-Graduação em Ciências Aplicadas à Hematologia, Universidade do Estado do Amazonas (UEA), Manaus, AM Brazil; 2 Diretoria de Ensino e Pesquisa, Fundação Hospitalar de Hematologia e Hemoterapia do Amazonas (HEMOAM), Av. Constantino Nery, 4397, Chapada, Manaus, AM 69050-001 Brazil; 3 Programa de Pós-Graduação em Imunologia Básica e Aplicada, Instituto de Ciências Biológicas, Universidade Federal do Amazonas (UFAM), Manaus, AM Brazil; 4 Rede Genômica de Vigilância em Saúde do Amazonas (REGESAM), Manaus, AM Brazil; 5 Laboratório de Micobacteriologia, Instituto Nacional de Pesquisas da Amazônia (INPA), Manaus, AM Brazil; 6 Programa de Pós-Graduação em Medicina Tropical, Universidade do Estado do Amazonas (UEA), Manaus, AM Brazil; 7 Instituto de Pesquisa Clínica Carlos Borborema, Fundação de Medicina Tropical Doutor Heitor Vieira Dourado (FMT-HVD), Manaus, AM Brazil; 8 Escola de Enfermagem de Manaus, Universidade Federal do Amazonas (UFAM), Manaus, AM Brazil

**Keywords:** Immunogenetics, Medical genetics, Genetics, Immunology, Immunogenetics, Inflammation, Innate immunity

## Abstract

The immune system plays an important role in the control of cancer development. To investigate the possible association of inflammasome genes to childhood leukemia we performed a case-control study with 158 patients with acute lymphoblastic leukemia and 192 healthy individuals. The *IL1B* and *IL18* genetic polymorphisms were genotyped by Polymerase Chain Reaction-Restriction Fragment Length Polymorphism (PCR-RFLP) and *NLRP1, NLRP3* and *P2RX7* were genotyped using Real Time quantitative PCR (qPCR). The *IL1B C/T rs19644* genotype was associated with the risk of developing ALL (C/C vs. C/T + T/T OR: 2.48 [95% CI: 1.26–4.88, *p* = *0.006*]; C/C vs C/T OR: 2.74 [95% CI: 1.37–5.51, *p* = *0.003*]) and the *NLRP1 A/T rs12150220* (OR: 0.37 [95% CI: 0.16–0.87, *p* = *0.023*]) was associated with protection against infectious comorbidities. It was not found association between *NLRP3* and *P2RX7* polymorphisms and acute lymphoblastic leukemia in our study. Our results suggest that the inflammasome single-variant polymorphisms (SNVs) may play a role in the development and prognostic of childhood leukemia. However, this finds requires further study within a larger population in order to prove it.

## Introduction

Acute lymphoblastic leukemia (ALL) is a hematopoietic neoplasm characterized by the exacerbated proliferation of blasts in bone marrow and affects mainly children aged 2 to 15 years old. In Brazil, according to the National Cancer Institute (INCA), it is estimated that for each year of the 2020–2022 triennium, there will be 5920 new cases of leukemia (Acute and chronic) in men and 4860 in women in Brazil, which corresponds to an estimated risk of 5.67 new cases per 100 thousand men and 4.56 for each 100 thousand women^1,2^.

Due to its unknown etiology, studies associate the manifestation of ALL with the interaction of genetic and environmental factors, however, less than 10% of cases are attributed to heredity^3,4^. New evidence indicates that inflammation plays an important role in all stages of cancer development. Since inflammation promotes the accumulation of genetic alterations that can inhibit the cell death control pathways of hematopoietic progenitor stem cells (HSPCs) and contribute to the generation of pre-leukemic clones. Mel Greaves observed that a low stimulation of the immune system in early childhood followed by a second response to infections, could be responsible for the dysregulation of the immune system and increase the chances of developing ALL^5^. However, the process for the development of ALL remains unknown^6^.

The inflammasome complex constitutes components of innate immunity involved in inflammatory processes and has been associated with the development of autoimmune inflammatory diseases and several types of cancers^7,8^. In acute lymphoblastic leukemia, NLRP1a-induced pyroptosis in hematopoietic progenitor cells can prevent cell proliferation and differentiation, contributing to the proliferation of altered blasts that will trigger the disease. The dysregulation of the inflammasome complex can also influence the prognosis of patients, since studies report that the constitutive activation of *NLRP3* seems to cleave the glucocorticoid receptor, this being the first line of treatment for ALL, and thus increase the number of relapses^9,10^. Besides, studies reported that the genetic variants of inflammasome related genes contribute to ALL pathogenesis and prognosis since *CARD8* rs2043211 A/T and T/T genotypes were associated with susceptibility, lower white blood cell (WBC) count and T-cell immunophenotype. *NF-κB-94 ins/del ATTG* was associated with protection in susceptibility of ALL. In addition, *IL1β* rs16944 and *IL18* rs1946518 seems to increase the mRNA expression of *NLRP3* and secretion of downstream cytokines^11^.

Although inflammasomes are associated with several types of diseases, there are few studies that demonstrate the relationship between the SNVs involving *IL1B* (Interleukin 1 beta)*, IL18* (Interleukin 18)*, NLRP1* (NLR family pyrin domain containing 1), *NLRP3* (NLR family pyrin domain containing 3) and *P2RX7* (Purinergic receptor P2X7) genes and their susceptibility or influence on the prognosis of ALL patients. In this study, we described that variants of inflammasomes were associated with the risk of developing pediatric ALL, and the resulting clinical prognosis in these patients.

## Results

### Clinical and epidemiological baseline of the patients

Demographic, clinical and laboratory data of ALL patients and controls are shown in Table 1. The mean age among individuals in the control group and patients with ALL was 38 and 12, respectively. In addition, the male gender was predominant in both groups (66% and 63%). The immunophenotype B-ALL was predominant (85%) in this study. Regarding the presence of comorbidities, 45% had some type of comorbidity on diagnosis, the most frequent being infectious diseases (86%) (e.g., cytomegalovirus, toxoplasmosis, rubella, varicella, parasitic diseases, among others), followed by other comorbidities (14%) (e.g., Aplasia and Burkitt's lymphoma). Most patients relapsed during treatment (66%) and approximately 41% of ALL patients died during treatment. The hemoglobin average was 8.65 g/dL, hematocrit 25.4 g/dL, leukocytes 4.720/mm^3^ and platelets 39.000/mm^3^.

**Table 1:** Selected characteristics of the childhood leukemia and controls in the Brazilian Amazon.

Variables	Healthy individuals	ALL cases
	(n = 192)	(n = 158)
**Age** (years, median [IQR])^a^	38 [26–52]	12 [4–17]
**Gender**		
Male, n (%)	127 (66%)	100 (63%)
Female, n (%)	65 (34%)	58 (37%)
**Ethnicity**		
White	40 (21%)	21 (13%)
Admixed	139 (72%)	135 (86%)
Black	7 (4%)	–
Indian	4 (2%)	–
Yellow	2 (1%)	(1%)
**Immunophenotype**		
B-ALL	–	135 (85%)
T-ALL	–	23 (15%)
**Residence**		
Manaus	192 (100%)	90 (57%)
Interior of Amazonas	–	57 (36%)
Other state	–	11 (7%)
**Comorbidities**		
Yes, n (%)	–	72 (45%)
Infectious diseases, n (%)	–	62 (86%)
Others, n (%)	–	10 (14%)
No, n (%)	–	86 (55%)
**Relapse**		
Yes, n (%)	–	105 (66%)
No, n (%)	–	53 (44%)
**Death**		
Yes, n (%)	–	66 (41%)
No, n (%)	–	92 (59%)
**Hemoglobin** (g/dL, median [IQR])^a^	–	8.65 [6.5–10.3]
**Hematocrit** (g/dL, median [IQR]) ^a^	–	25.4 [20.6–30.8]
**Leukocyte** (g/dL, median [IQR]) ^a^	–	4.720 [2.110–39.200]
**Platelets** (g/dL, median [IQR]) ^a^	–	39.000 [16.225–126.500]

^a^g/dL = Gram per decilitre. IQR = Interquantile Range.

### Association of IL1B C/T rs19644 genotype with pediatric acute lymphoblastic leukemia

In Table 2, it is possible to observe the genotypic frequencies of all the SNVs under study. Among all the SNVs, *IL1B rs19644* (*p* =  ≤ *0.001*), *P2RX7 rs2230911* (*p* = *0.042*), *NLRP1* rs35865013 (*p* = *0.000*) deviated from the Hardy–Weinberg balance. The *IL1B C/T rs19644* genotype appears to be a risk factor for the development of ALL (C/C vs. C/T + T/T OR: 2.48 [95% CI: 1.26–4.88, *p* = *0.006*]; C/C vs. C/T OR: 2.74 [95% CI: 1.37–5.51, *p* = *0.003*]. This it is also observed when it adjusted for age and sex (OR: 2.48 [95% CI: 1.14–5.40, *p* = *0.*001]). Supplementary Table S1 summarizes the results from multivariate regression analysis for all the SNVs with acute lymphoblastic leukemia.

**Table 2:** Multivariate analysis adjusted for sex and age for the association of single-variant polymorphisms (SNVs) in study with acute lymphoblastic leukemia.

Genetic models	Controls n = 192 (%)	ALL cases n = 158 (%)	OR (95% CI)	*p* value	AIC	OR (95% CI) adj	*p* value adj	AIC
***IL1B rs169744***								
**Codominant**								
CC	35 (0.18)	13 (0.08)						
CT	95 (0.49)	97 (0.61)	2.74 (1.37–5.51)	***0.003***	478.9	2.48 (1.14–5.40)	***0.001***	236.3
TT	62 (0.33)	48 (0.31)	2.08 (0.99–4.36)	***0.049***		0.55 (0.20–1.49)		
**Dominant**								
TT	62 (0.32)	48 (0.30)	1.09 (0.69–1.72)	0.701	485.7	1.66 (0.81–3.40)	0.164	245.4
CT-CC	130 (0.68)	110 (0.70)						
**Recessive**								
TT-CT	157 (0.82)	145 (0.92)						
C/C	35 (0.18)	13 (0.08)	0.40 (0.20–0.79)	***0.005***	478.3	0.31 (0.13–0.74)	0.006	239.8
**Overdominant**								
TT-CC	97 (0.51)	61 (0.39)	1.62 (1.06–2.49)	***0.025***	480.9	3.13 (1.59–6.19)	***0.000***	235.7
CT	95 (0.49)	97 (0.61)						
Log-Additive 0,1,2	192 (0.55)	158 (0.45)	0.82 (0.59–1.14)	0.244	484.5	0.86 (0.53–1.37)	0.521	246.9

Adjusted for sex and age (*p* value_adj_, OR_adj_); OR: Odds Ratio; *p* value: < 0.05; 95% confidence interval; AIC: Akaike information criterion value.

### The NLRP1 A/T rs12150220 genotype is associated with protection against infectious comorbidities in pediatric ALL patients

In Table 3, it is possible to observe the genotype frequency of the SNVs under study in relation to comorbidities (infectious diseases), relapse and death. Logistic regression analysis was performed in order to investigate the association of genotypes with the variables under study. The *NLRP1 A/T rs12150220* (OR: 0.37 [95% CI: 0.16–0.87, *p* = *0.023*) was associated with protection against infectious comorbidities and this it also observed in multivariate analysis adjusted for age and sex (OR: 0.34 [95% CI: 0.16–0.73, *p* = *0.003*). In this study, it was not observed association to relapse and death. Supplementary Table S2 summarizes the results from multivariate regression analysis for all the SNVs according to infectious comorbidities, relapse and death.

**Table 3:** Multivariate analysis adjusted for sex and age for the association of single-variant polymorphisms (SNVs) in study with infectious comorbidities in acute lymphoblastic leukemia patients.

Genetic models	Infectious comorbidities							
	No n = 86 (%)	Yes n = 62 (%)	OR (95% CI)	*p *value	AIC	OR (95% CI) adj	*p* value adj	AIC
***NLRP1 rs12150220***								
**Codominant**								
AA	29 (0.34)	33 (0.53)						
AT	50 (0.58)	18 (0.29)	0.32 (0.15–0.66)	***0.001***	194.3	0.34 (0.16–0.73)	***0.003***	189.8
TT	7 (0.08)	11 (0.18)	1.38 (0.47–4.03)			1.54 (0.50–4.72)		
**Dominant**								
AA	29 (0.34)	33 (0.53)	0.45 (0.23–0.87)	***0.017***	199.6	0.49 (0.24–0.97)	***0.039***	194.8
AT-TT	57 (0.66)	29 (0.47)						
**Recessive**								
AA-AT	79 (0.92)	51 (0.82)						
AT	7 (0.08)	11 (0.18)	2.43 (0.89–6.69)	0.079	202.2	2.58 (0.89–7.48)	0.074	195.8
**Overdominant**								
AT-TT	36 (0.42)	44 (0.71)	0.29 (0.15–0.59)	***0.000***	192.7	0.31 (0.15–0.64)	***0.001***	188.3
AT	50 (0.58)	18 (0.29)						
log-Additive 0,1,2	86 (0.58)	62 (0.42)	0.80 (0.49–1.31)	0.375	204.5	0.85 (0.51–1.40)	0.521	198.6

Adjusted for sex and age (*p* value_adj_, OR_adj_); OR: Odds Ratio; *p* value: < 0.05; 95% confidence interval; AIC: Akaike information criterion value.

The case and control groups showed similar proportions of allele frequencies for each SNVs, and thus, no significant difference in allelic frequencies was found between both groups. The major allele frequencies for each polymorphism are shown in Supplementary Table S3.

## Discussion

Inflammasomes are multimeric molecular complexes, formed in the cytoplasm in response to endogenous and exogenous stimuli that promote the activation of inflammatory caspases^12^. Over the years, inflammasomes have been linked to autoimmune^13,14^ and inflammatory diseases^15^ as well as several types of cancer^16^. Genetic variants of inflammasome related genes can contribute to ALL pathogenesis and prognosis as CARD8 and NF-κB^11^, however, there are few studies that demonstrate the role of others genes involved in pathway inflammasome in ALL.

To investigate the possible genetic contribution to childhood leukemia in the inflammasomes, we performed a study for the SNVs related to inflammasome genes. When the frequency of SNVs in the case and control group with other populations in the world was evaluated, we observed that all subjects presented sequences similar to the frequencies described for the Native South-American population, except *NLRP1 rs35865013 A/G* (A: 0.402 and G: 0.598), which presented a similar frequency to the population of South Asia^17^. The population of the Amazon Region has the highest high degree of inter-ethnic admixture due to the intense miscegenation process that occurred in the region and the strong indigenous influence on the population. Children with admixed ancestry have a higher risk of developing ALL due to the existence of genetic variations characteristic of Native South American. Native South American ancestry is predominantly found in the northern region of Brazil, where approximately 80% of the Amazon region is located^18^, and the literature has already described its association with susceptibility to ALL in children in the Brazilian Amazon^19^.

In our study, the *IL1B rs16944* polymorphism was associated with the risk of developing ALL. The *IL1B* gene is located on chromosome 2q14 and contains many single-nucleotide variants. *IL1B rs16944* is located in the promoter region and T allele is associated with the increased of transcription activity and production of IL-1β cytokine^20–22^ and increased mRNA expression of *NLRP3* and *ASC*^11^. In the literature, this polymorphism has been associated with susceptibility or worse prognosis in individuals with autoimmune diseases and in several types of cancers^21,23^. In a study by Yin et al. [2016], it was shown that individuals with *IL1B G/G rs16944* genotype are at risk of developing Myelodysplastic Syndrome (MDS)^24^. In addition, polymorphisms involving the IL-1β cytokine were associated with cytogenetic assessment of what would be considered a good prognosis in patients with acute myeloid leukemia (AML) (*p* = *0.043*)^25^.

After infection or injury, an IL-1β is found at high medullary levels by monocytes and endothelial cells, and promotes myeloid differentiation through activation of the NF-kB pathway that results in the expansion of HSPCs^26^. Chronic exposure to IL-1β significantly impairs self-renewal and the ability of HSPCs to differentiate into lymphoid and erythroid lineage ^27^. Therefore, chronic sustained inflammation may elicit the stem cell insult by inducing a state of chronic oxidative stress with elevated levels of reactive oxygen species (ROS) in the bone marrow, thereby creating a high-risk microenvironment for induction of genetic alterations due to the persistent inflammation-induced oxidative damage to DNA in hematopoietic cells ^28^.

Chronic immune stimulation from infectious processes is a trigger for AML and MDS. The history of infectious diseases (tuberculosis, intestinal diseases, pneumonia, septicemia, pyelonephritis, sinusitis, nasopharyngitis, hepatitis C, cytomegalovirus infection and upper respiratory tract infection) was associated with 1.3 times more chances of developing AML or MDS, even if the infection occurred 3 years before the onset of the disease^29^. Thus, chronic inflammation can be identified as one of the triggers of hematological neoplasms.

IL-18 is an important cytokine resulting from NLRP3 inflammasome activation, which is involved in the innate and acquired immune response. In multiple myeloma, the increase in serum IL-18 is associated with disease progression and lower chances of patient survival^30^. In this study, no association was found between *IL18* polymorphism and ALL. However, in the study by Yalçin et al. on the Turkish population (2014), the G/C and C/C genotypes were associated with the risk of developing chronic myeloid leukemia, and the C/C genotype was associated with the risk of developing chronic lymphoid leukemia, which suggests a relationship between this polymorphism and the development of chronic leukemias characterized by the proliferation of mature cells, however, with loss of functionality^31^.

The *NLRP1* inflammasome is expressed in hematopoietic progenitor cells and its activation results in a process of cell death which is dependent on Caspase 1 and is called pyroptosis. Some studies report that the prolonged cytopenia, induced by the activation of NLRP1 during infectious processes, ensures a proliferative advantage for the leukemic clone, as suggested by the Mel Greaves hypothesis on the development of ALL^32,33^. In chronic myeloid leukemia (CML), overexpression of *NLRP1* gene is associated with the promotion of proliferation and reduction of apoptosis in CML cells, in addition to inducing resistance to imatinib^34^.

In this study, the *NLRP1 A/T rs12150220* genotype was associated with protection against infectious diseases. In ALL, infections are present in 49% of patients on diagnosis^35^. Studies report that susceptibility to congenital toxoplasmosis is significantly associated with SNVs and involves the locus of the *NLRP1* gene^36^, which strengthens the Mel Greaves hypothesis that genetic changes in the uterus followed by the acquisition of infections by common pathogens are involved in the development of ALL^5,37^.

NLRP3 is currently the best-studied member of the inflammasome family expressed in hematopoietic and lymphopoietic cells being responsible for the migration and spread of leukemic cells. The inflammatory process in leukemic patients promotes release of several chemoattractants and thus increases trafficking of leukemic cells and their spread within hematopoietic organs that contribute for the ALL development^38^. Paugh et al. (2016) reported that decreased methylation of the Caspase-1 promoter results in increased transcription and activation of *NLRP3* and *Caspase1*, which cleaves the glucocorticoid receptors used in the treatment of ALL, what suggests their association with relapse episodes^10, 39^. Besides, the *NLRP3* expression is increased in patients with ALL compared to healthy individuals^40^ but decreased in CML, what suggests different roles of inflammasome activity in acute and chronic leukemia. The A/A genotype *NLRP3* rs35829419 was associated with risk of acute myeloid leukemia^25^ but not when it observed CML patients^41^. In this study, we did not find association of *NLRP3* SNVs, futher studies are necessary to better elucidate their role of NLRP3 in ALL.

The P2RX7 receptor is responsible for making NLRP3 sensitive to ATP, which is one of the main DAMPs released during inflammation and is highly expressed in tumor cells. Studies describe the high expression of *P2RX7* in samples from ALL patients, especially those who relapse, as well as an association with dysregulation of the HSPCs’ normal functioning, since it affects the ability of colony formation in vitro, which impairs the clonal expansion process observed in the HSPCs. However, the role of this mechanism in the development of ALL is still unknown ^42^.

The *P2RX7* polymorphisms cause a loss of receptor function, mainly in macrophages, and is responsible for a partial reduction of the channel and formation of pores in the membrane^43–45^. In chronic lymphoid leukemia, *P2RX7 rs3751143* polymorphism appears to influence patient survival, and the A/C genotype is associated with longer survival (104 months) than the A/A genotype (72 months)^46,47^. In this study, we did not find association between P2RX7 polymorphisms and ALL. However, one of the main infectious comorbidities found in our patients was toxoplasmosis, an infection caused by *Toxoplasma gondii*. The P2RX7 receptor is an important mediator in the control of infection by *Toxoplasma gondii*, since it prevents its proliferation by stimulating the production of reactive oxygen species (ROS) and facilitates the acidification of parasitophorous vacuoles in macrophages infected by the parasite^48^. Loss of receptor function may be responsible for the susceptibility to *Toxoplasma gondii* infection in patients with acute lymphoblastic leukemia in this study, however, prospective studies are needed to confirm this relationship.

This study has some limitations. Despite the association of SNVs with ALL inflammasome and clinical data, the study population is small compared to other studies involving SNVs. Thus, prospective studies with a larger population are necessary in order to confirm the importance of the polymorphisms under study in ALL, including those in adult patients and rearrangements. The small sample size did not allow the comparison of the studied genotypes and alleles with the laboratory data. The absence of the analysis of gene expression and the determination of serum levels of proteins also limited the study, since they made it impossible to gain a better understanding of the influence of these molecules on the variables under study. Moreover, the control group was composed of blood donors. According to the Brazilian Ministry of Health, candidates of at least 16 years of age with the requirements of those responsible are eligible, however, the group with the highest adherence to donation are those over the age of 18, and these are the main components of our sample. However, the case group was composed of leukemic patients within the period of major incidence of disease (< 18 years). Although we have included this topic as a limitation of the study, we understand that when using children as a control group, false results could be formulated, since they could develop the disease after the study. While adults, supposedly would have had a longer time to develop the disease.

To our knowledge, this is the first study to describe the frequency of polymorphisms in the inflammasome genes in patients with acute lymphoblastic leukemia in the Amazon region. Inflammasomes are important complexes in the defense of the host and contribute to neoplastic development. Thus, the variant *IL1B C/T rs16944* was associated with susceptibility to ALL in individuals from the Brazilian Amazon region. In addition, the variant *NLRP1 A/T rs12150220* can promove protection from infectious diseases in acute lymphoblastic leukemia patients. However, future studies should be carried out in order to better elucidate the influence of these SNVs on the pathogenesis of ALL.

## Materials and methods

### Patients and sampling

In the case group, samples were included from 158 pediatric patients diagnosed with ALL according to the classification criteria of the World Health Organization (WHO)^49^, and who were treated at Fundação Hospitalar de Hematologia e Hemoterapia do Amazonas (HEMOAM). The patients had cryopreserved samples in the DNA library of the HLA typing laboratory of the HEMOAM, were < 18 years, of either gender or unrelated. Insufficient or low-concentration DNA samples, and patients with a history of bone marrow transplantation were excluded from the study.

The healthy individuals (control group) consisted of 192 samples from blood donors of either gender, who donated at HEMOAM between January and December 2015 and agreed to participate in the research. In order to be considered healthy, all candidates were tested serologically for HIV, HCV, HBV, HBV, HTLV-1/2, Syphilis and Chagas disease and for HIV, HBV, HCV using the NAT HIV/HCV/HBV Kit. In addition, they were screened in interviews for diseases and other risk factors, according to the Brazilian Ministry of Health technical standards. Insufficient or low-concentration DNA samples and related candidates were excluded.

### Ethical issues

This study was approved by the Research Ethics Committee of the HEMOAM Foundation under protocol number 3.335.123/2019, CAAE 12615918.9.0000.0009. Prior inclusion of all patients and controls in the study, all the respective parents or legal guardians read and signed the informed consent form. This study was carried out in accordance with the guidelines of the Declaration of Helsinki and Resolution 466/12 of the Brazilian National Health Council for research involving human beings.

### Biological sample and data collection

Approximately 4 mL of peripheral blood was obtained from ALL patients in remission using venipuncture in tubes with a vacuum system containing EDTA (BD Vacutainer EDTA K2®). From the control group, approximately 12 mL of peripheral blood were collected by venipuncture in tubes with a vacuum system containing EDTA, Sodium Citrate (BD Vacutainer Citrate Tube®) and with Separator Gel (Gel BD SST® II Advance) for complete blood count, biochemical tests and serology, respectively. In addition, demographic (age, gender), laboratory (blood count, immunophenotype) and clinical (comorbidities [infectious diseases and others], relapse and death), data were obtained from searches of medical records in the medical and statistical care system (SAME), iDoctor system and statistics sector of the HEMOAM.

Infections serologically tested as IgG^+^ and IgM^+^ (cytomegalovirus, toxoplasmosis, rubella, varicella, parasitic diseases, among others) were considered as infectious comorbidities according to Silva-Júnior et al. (2019)^50^. Aplasia, Systemic Arterial Hypertension (SAH), Diabetes Mellitus and Down Syndrome were included in the group “Others”. In addition, patients who relapsed after induction therapy (35th day of treatment) were used as a relapse criterion. Death that occurred within 5 years after diagnosis was considered.

### Genomic DNA extraction

Genomic DNA extraction from blood samples (case group) was performed with the triplePrep Kit® GenomicPrep DNA Extraction kit (GE Healthcare Life Sciences) and BIOPUR Kit mini spin plus extraction® (Mobius Life Sciences) following the recommendations described by the manufacturer. For the samples of the control group, the QIAmp DNA Kit (QIAGEN, Chatsworth, CA, USA) was used. After extraction, the DNA was evaluated by readings at 260 nm with the NanoDrop™ 2000/2000c spectrophotometer (Thermo Scientific™).

### Selection and genotyping by PCR-RFLP

Candidate gene regions were selected based on SNP databases (Cancer Genome Anatomy Project and SNP500 database)^51^. Among them, we selected upstream (*IL1B* rs16944 and *IL18* rs187238), downstream (*NLRP3* rs10754558 and rs10802502), missense (*NLRP1* rs12150220, *P2RX7* rs3751143 and rs2230911) and intron variants (*NLRP1* rs35865013). SNVs based upon following criteria: functional effects, minor allele frequency (MAF) (≥ 3%) and previously reported association with hematological neoplasm (mainly leukemia).

PCR reactions were performed according to the protocol described by Bhat et al. (2014) and Folwaczny et al. (2005) and the methods description partly reproduces their wording^52, 53^. For *IL1B rs16944,* the following sequences were used: 5′TGGCATTGATCTGGTTCATC-3′ (Forward) and 5′GTTTAGGAATCTTCCCACTT-3′ (Reverse); and for *IL18 rs187238*: 5′CACAGAGCC CCAACTTTTTACGGGTAGA-3′ (Forward) and 3′GACTGCTGTCGGCACTCCTTGG-5′ (Reverse). The mix was composed of 17.3 μL of H2O MiliQ, 2.5 μL of 10 × buffer, 2.0 μL of MgCl2, 1.0 μL of dNTPs, 0.5 μL of each primer, 0.2 μL of Taq DNA polymerase and 2.0 μL of Genomic DNA. Clicking was performed on the Applied Biosystems thermocycler (Veriti® 96-Well Thermal Cycler, Carlsbad, USA) using the following programs: 1 cycle at 95 °C for 4 min, 35 cycles at 95 °C for 30 s, 56 °C for 30 s, 72 °C for 30 s and 72 °C for 10 min (*IL1B*) and 1 cycle at 95 °C for 10 min, 35 cycles at 95 °C for 30 s, 60 °C for 30 s, 72 °C for 30 s and 72 °C for 10 min (*IL18*). The PCR product was subjected to the RFLP reaction with 7.8 μL of H2O MiliQ, 2.0 μL of buffer 4, 0.2 μL of the restriction enzyme *IL1B rs16944* (*AvaI*) and *IL18 rs187238* (*MboII*) (10 U/μL, Promega, Madison WI, USA) and 15.0 μL of the PCR reaction product, with subsequent incubation in a thermoblock at 37 °C overnight (~ 16 h). The digestion of the products was observed in a 3% agarose gel, and the genotyping was characterized by *IL1B* (T/T-304 bp), (C/C-190 pb), (C/T-304/190/114 bp) and *IL18* (C/C: 155 bp), (G/G: 116 bp) and (G/C: 155/116/39).

### Genotyping by real-time quantitative PCR (qPCR)

The genotyping of the *NLRP1, NLRP3* and *P2RX7* polymorphisms was performed using the Real-Time Quantitative PCR (qPCR) technique, using allele specific TaqMan fluorescent probes that allow the discrimination of the studied SNVs. The qPCR reactions were performed in 96-well microplates with 2.25 µL of ultrapure water, 2.5 µL of the genotyping Master Mix (1×), 0.25 μL of TaqMan® assay (20×), containing 36 μM of each primer and 8 μM of each TaqMan® probe with a final volume of 7 μL. The probes used in experiment are shown in Supplementary Table S4. The QuantiStudio™ Design & Analysis Applied Biosystems thermal cycler was used to amplify the sequences of interest and allelic discrimination under the following condition: 95 °C for 10 min for activation; 40 cycles at 92 °C for 15 s for denaturation; and 40 cycles at 60 °C for 90 s for annealing and extension.

### Statistical and data analysis

Association between the SNVs and ALL susceptibility was calculated by a Fisher’s exact test with a 95% confidence interval (95% CI) using the software GraphPad Prism v.5 (San Diego, CA, USA). Alleles analysis was performed via the website http://ihg.gsf.de/cgibin/hw/hwa1.pl. and the associations between the allelic/genotype frequencies among patients according to infectious comorbidities, relapse and death were examined under four genetic models, specifically, codominant, dominant, recessive and overdominant models using the package “SNPassoc” version 2.0.2 (https://cran.r-project.org/web/packages/SNPassoc/index.html) for R software version 4.0.3 (www.r-project.org). The best genetic model was performed via Akaike information criterion (AIC). The Hardy–Weinberg (HW) balance was determined for all the SNVs. An univariate and multivariate logistic regression analysis was performed in order to investigate the association of genotypes with the presence of infectious comorbidities, relapse and death using the software STATA v.13 (Stata Corp, 2013, College Station, Texas, USA) after adjusting for age and sex.

## Supplementary Information

Supplementary Information.
