# Supplementary material for: Genetic polymorphisms of inflammasome genes associated with pediatric acute lymphoblastic leukemia and clinical prognosis in the Brazilian Amazon
Source: Sci Rep. 2021 May 10;11:9869. doi: 10.1038/s41598-021-89310-4 (PMC8110953; doi:10.1038/s41598-021-89310-4)
Supplement: Supplementary file 1 — Supplementary Information. [file 41598_2021_89310_MOESM1_ESM.docx]

**SUPPLEMENTARY MATERIALS**

**Supplementary Table S1.** Multivariate analysis adjusted for sex and age for the association of single-variant polymorphisms (SNVs) in study with acute lymphoblastic leukemia.

| Genetic  models | Controls  n=192 (%) | ALL cases  n=158 (%) | OR (95% Cl) | *p* value | AIC | OR (95% CI)  adj | *p* value  adj | AIC |
| --- | --- | --- | --- | --- | --- | --- | --- | --- |
|  | ***IL18* rs187238** | | | | | |  |  |
| Codominant |  |  |  |  |  |  |  |  |
| GG | 82 (0.43) | 67 (0.42) |  |  |  |  |  |  |
| GC | 90 (0.47) | 77 (0.48) | 1.05 (0.67 – 1.63) | 0.868 | 487.6 | 1.26 (0.64 – 2.48) | 0.764 | 248.8 |
| CC | 20 (0.10) | 14 (0.10) | 0.86 (0.40 – 1.82) |  |  | 1.32 (0.42 – 4.11) |  |  |
| Dominant |  |  |  |  |  |  |  |  |
| GG | 82 (0.43) | 67 (0.42) | 1.01 (0.66 – 1.55) | 0.954 | 485.9 | 1.27 (0.67 – 2.42) | 0.466 | 246.8 |
| GC-CC | 110 (0.57) | 91 (0.58) |  |  |  |  |  |  |
| Recessive |  |  |  |  |  |  |  |  |
| GG-GC | 172 (0.90) | 144 (0.91) |  |  |  |  |  |  |
| CC | 20 (0.10) | 14 (0.09) | 0.84 (0.41 – 1.71) | 0.623 | 485.7 | 1.17 (0.40 – 3.45) | 0.774 | 247.2 |
| Overdominant |  |  |  |  |  |  |  |  |
| GG-CC | 102 (0.53) | 81 (0.51) | 1.08 (0.71 – 1.64) | 0.728 | 485.8 | 1.20 (0.63 – 2.28) | 0.579 | 247.0 |
| GC | 90 (0.47) | 77 (0.49) |  |  |  |  |  |  |
| Log-Additive  0,1,2 | 192 (0.55) | 158 (0.45) | 0.97 (0.70 – 1.35) | 0.856 | 485.9 | 1.19 (0.73 – 1.95) | 0.490 | 246.8 |
|  | ***P2RX7* rs2230911** | | | | | |  |  |
| Codominant |  |  |  |  |  |  |  |  |
| CC | 128 (0.66) | 97 (0.61) |  |  |  |  |  |  |
| CG | 57 (0.30) | 59 (0.37) | 1.37 (0.87 – 2.14) | 0.138 | 483.9 | 1.85 (0.92 – 3.69) | 0.098 | 244.7 |
| GG | 7 (0.04) | 2 (0.02) | 0.38 (0.08 – 1.86) |  |  | 0.25 (0.02 – 3.91) |  |  |
| Dominant |  |  |  |  |  |  |  |  |
| CC | 128 (0.67) | 97 (0.61) | 1.26 (0.81 – 1.95) | 0.305 | 484.8 | 1.65 (0.84 – 3.24) | 0.140 | 245.1 |
| CG-GG | 64 (0.33) | 61 (0.39) |  |  |  |  |  |  |
| Recessive |  |  |  |  |  |  |  |  |
| CC-CG | 185 (0.96) | 156 (0.99) |  |  |  |  |  |  |
| GG | 7 (0.04) | 2 (0.01) | 0.34 (0.07 – 1.65) | 0.147 | 483.8 | 0.20 (0.01 – 3.15) | 0.211 | 245.7 |
| Overdominant |  |  |  |  |  |  |  |  |
| CC-GG | 135 (0.70) | 99 (0.63) | 1.41 (0.90 – 2.21) | 0.130 | 483.6 |  |  |  |
| CG | 57 (0.30) | 59 (0.38) |  |  |  | 1.92 (0.96 – 3.82) | 0.061 | 243.8 |
| Log-Additive  0,1,2 | 192 (0.56) | 158 (0.44) | 1.11 (0.75 – 1.64) | 0.615 | 485.6 | 1.36 (0.75 – 2.46) | 0.317 | 246.3 |
|  | ***P2RX7* rs3751143** | | | | | |  |  |
| Codominant |  |  |  |  |  |  |  |  |
| AA | 127 (0.66) | 91 (0.57) |  |  |  |  |  |  |
| AC | 59 (0.31) | 62 (0.39) | 1.47 (0.94 – 2.29) | 0.242 | 485.1 | 1.62 (0.80 – 3.27) | 0.318 | 247.0 |
| CC | 6 (0.03) | 5 (0.04) | 1.16 (0.34 – 3.93) |  |  | 0.65 (0.12 – 3.61) |  |  |
| Dominant |  |  |  |  |  |  |  |  |
| AA | 127 (0.66) | 91 (0.58) | 1.44 (0.93 – 2.22) | 0.100 | 483.2 | 1.47 (0.75 – 2.89) | 0.257 | 246.0 |
| AC-CC | 65 (0.44) | 67 (0.42) |  |  |  |  |  |  |
| Recessive |  |  |  |  |  |  |  |  |
| AA-AC | 186 (0.97) | 153 (0.97) |  |  |  |  |  |  |
| CC | 6 (0.03) | 5 (0.03) | 1.01 (0.30 – 3.38) | 0.983 | 485.9 | 0.56 (0.10 – 3.04) | 0.500 | 246.8 |
| Overdominant |  |  |  |  |  |  |  |  |
| AA-CC | 133 (0.69) | 96 (0.61) | 1.46 (0.94 – 2.27) | 0.096 | 483.1 | 1.66 (0.83 – 3.32) | 0.152 | 245.2 |
| AC | 59 (0.31) | 62 (0.39) |  |  |  |  |  |  |
| log-Addtive  0,1,2 | 192 (0.55) | 158 (0.45) | 1.33 (0.90 – 1.94) | 0.147 | 483.8 | 1.25 (0.70 – 2.23) | 0.452 | 246.7 |
|  | ***NLRP1* rs35865013** | | | | | |  |  |
| Codominant |  |  | |  |  |  |  |  |
| AA | 28 (0.14) | 29 (0.18) |  |  |  |  |  |  |
| AG | 161 (0.84) | 129 (0.82) | 0.77 (0.44 – 1.37) | 0.247 | 483.5 | 0.71 (0.28 – 1.76) | 0.333 | 247.1 |
| GG | 3 (0.02) | 0 | - | - | - | - | - | - |
| Dominant |  |  |  |  |  |  |  |  |
| AA | 28 (0.15) | 29 (0.18) | 0.76 (0.43 – 1.34) | 0.342 | 485.0 | 0.70 (0.28 – 1.74) | 0.435 | 246.7 |
| AG-GG | 164 (0.85) | 129 (0.82) |  |  |  |  |  |  |
| Recessive |  |  |  |  |  |  |  |  |
| AA-AG | 189 (0.98) | 158 (100) |  |  |  |  | 0.200 | 245.6 |
| GG | 3 (0.02) | 0 | - | - | - | - |  |  |
| Overdominant |  |  |  |  |  |  |  |  |
| AA-GG | 31 (0.16) | 29 (0.18) | 0.86 (0.49 – 1.49) | 0.585 | 485.6 | 0.80 (0.33 – 1.94) | 0.620 | 247.0 |
| AG | 161 (0.83) | 129 (0.82) |  |  |  |  |  |  |
| log-Additive  0,1,2 | 192 (0.55) | 158 (0.45) | 0.70 (0.40 – 1.21) | 0.247 | 484.2 | 0.63 (0.26 – 1.54) | 0.310 | 246.3 |
|  | ***NLRP1* rs12150220** | | | | | |  |  |
| Codominant |  |  |  |  |  |  |  |  |
| AA | 80 (0.42) | 70 (0.44) |  |  |  |  |  |  |
| AT | 90 (0.46) | 70 (0.44) | 0.89 (0.57 – 1.39) | 0.875 | 487.6 | 0.87 (0.44 – 1.72) | 0.764 | 248.8 |
| TT | 22 (0.12) | 18 (0.12) | 0.94 (0.46 – 1.88) |  |  | 0.69 (0.24 – 1.93) |  |  |
| Dominant |  |  |  |  |  |  |  |  |
| AA | 80 (0.42) | 70 (0.44) | 0.90 (0.59 – 1.37) | 0.619 | 485.6 | 0.83 (0.44 – 1.58) | 0.565 | 247.0 |
| AT-TT | 112 (0.58) | 88 (0.56) |  |  |  |  |  |  |
| Recessive |  |  |  |  |  |  |  |  |
| AA-AT | 170 (0.88) | 140 (0.89) |  |  |  |  |  |  |
| AT | 22 (0.12) | 18 (0.11) | 0.99 (0.51 – 1.93) | 0.984 | 485.9 | 0.74 (0.28 – 1.95) | 0.536 | 246.9 |
| Overdominant |  |  |  |  |  |  |  |  |
| AT-TT | 102 (0.53) | 88 (0.56) | 0.90 (0.59 – 1.38) | 0.630 | 485.7 | 0.95 (0.50 – 1.80) | 0.865 | 247.3 |
| AT | 90 (0.47) | 70 (0.44) |  |  |  |  |  |  |
| log-Additive  0,1,2 | 192 (0.55) | 158 (0.45) | 0.94 (0.69 – 1.29) | 0.705 | 485.8 | 0.84 (0.52 – 1.35) | 0.471 | 246.8 |
|  | ***NLRP3* rs10754558** | | | | | |  |  |
| Codominant |  |  |  |  |  |  |  |  |
| CC | 116 (0.60) | 92 (0.58) |  |  |  |  |  |  |
| CG | 65 (0.34) | 56 (0.35) | 1.09 (0.69 – 1.70) | 0.911 | 487.7 | 0.98 (0.50 – 1.91) | 0.629 | 248.4 |
| GG | 11 (0.06) | 10 (0.10) | 1.15 (0.47 – 2.82) |  |  | 2.40 (0.37 – 15.41) |  |  |
| Dominant |  |  |  |  |  |  |  |  |
| CC | 116 (0.60) | 92 (0.58) | 1.09 (0.71 – 1.68) | 0.678 | 485.7 | 1.05 (0.55 – 2.01) | 0.872 | 247.3 |
| CG-GG | 76 (0.40) | 66 (0.42) |  |  |  |  |  |  |
| Recessive |  |  |  |  |  |  |  |  |
| CC-CG | 181 (0.94) | 148 (0.94) |  |  |  |  |  |  |
| GG | 11 (0.06) | 10 (0.06) | 1.11 (0.46 – 2.69) | 0.814 | 485.8 | 2.42 (0.38 – 15.24) | 0.337 | 246.4 |
| Overdominant |  |  |  |  |  |  |  |  |
| CC-GG | 127 (0.66) | 102 (0.65) | 1.07 (0.69 – 1.67) | 0.755 | 485.8 | 0.94 (0.49 – 1.81) | 0.844 | 247.2 |
| CG | 65 (0.34) | 56 (0.35) |  |  |  |  |  |  |
| log-Additive  0,1,2 | 192 (0.55) | 158 (0.45) | 1.08 (0.76 – 1.52) | 0.669 | 485.7 | 1.14 (0.65 – 2.01) | 0.653 | 247.1 |
|  | ***NLRP3* rs10805502** | | | | | |  |  |
| Codominant |  |  |  |  |  |  |  |  |
| CC | 73 (0.38) | 66 (0.42) |  |  |  |  |  |  |
| CT | 95 (0.50) | 76 (0.48) | 0.88 (0.56 – 1.39) | 0.680 | 487.1 | 0.90 (0.46 – 1.78) | 0.778 | 248.8 |
| TT | 24 (012) | 16 (0.10) | 0.74 (0.36 – 1.51) |  |  | 1.37 (0.41 – 4.63) |  |  |
| Dominant |  |  |  |  |  |  |  |  |
| CC | 73 (0.38) | 66 (0.42) | 0.86 (0.56 – 1.31) | 0.475 | 485.4 | 0.96 (0.50 – 1.85) | 0.899 | 247.3 |
| CT-TT | 119 (0.62) | 92 (0.58) |  |  |  |  |  |  |
| Recessive |  |  |  |  |  |  |  |  |
| CC-CT | 168 (0.88) | 142 (0.90) |  |  |  |  |  |  |
| TT | 24 (0.12) | 16 (0.10) | 0.79 (0.40 – 1.54) | 0.485 | 485.4 | 1.46 (0.46 – 4.60) | 0.518 | 246.9 |
| Overdominant |  |  |  |  |  |  |  |  |
| CC-TT | 97 (0.50) | 82 (0.52) | 0.95 (0.62 – 1.44) | 0.797 | 485.8 | 0.85 (0.45 – 1.62) | 0.627 | 247.1 |
| CT | 95 (0.50) | 76 (0.48) |  |  |  |  |  |  |
| log-Additive  0,1,2 | 192 (0.55) | 158 (0.45) | 0.87 (0.63 – 1.20) | 0.384 | 485.1 | 1.05 (0.63 – 1.76) | 0.846 | 247.3 |

Legend: Adjusted for sex and age (p value_ad_j, OR_adj_); OR: Odds Ratio; p value: < 0.05; 95% confidence interval; AIC: Akaike information criterion value.

**Supplementary Table S2.** Multivariate analysis adjusted for sex and age for the association of single-variante polymorphisms (SNVs) in study with infectious comorbidities in acute lymphoblastic leukemia patients.

|  | Infectious comorbidities | | | | | | | |
| --- | --- | --- | --- | --- | --- | --- | --- | --- |
| **Genetic**  **models** | **No**  **n=86 (%)** | **Yes**  **n=62 (%)** | **OR (95% CI)** | ***p* value** | **AIC** | **OR (95% CI)**  **adj** | ***p* value**  **adj** | **AIC** |
| ***IL1B* rs169744** | | | | | | | | |
| Codominant |  | | |  |  |  |  |  |
| TT | 30 (0.35) | 17 (0.27) |  |  |  |  |  |  |
| CT | 50 (0.58) | 39 (0.63) | 1.38 (0.66 – 2.85) | 0.577 | 206.2 | 1.65 (0.77 – 3.56) | 0.392 | 199.1 |
| CC | 6 (0.7) | 6 (0.10) | 1.76 (0.49 – 6.34) |  |  | 1.81 (0.49 – 6.63) |  |  |
| Dominant |  |  |  |  |  |  |  |  |
| TT | 30 (0.35) | 17 (0.27) | 1.42 (0.70 – 2.89) | 0.333 | 204.3 | 1.67 (0.79 – 3.54) | 0.173 | 197.2 |
| CT-CC | 56 (0.65) | 45 (0.73) |  |  |  |  |  |  |
| Recessive |  |  |  |  |  |  |  |  |
| TT-CT | 80 (0.93) | 56 (0.90) |  |  |  |  |  |  |
| CC | 6 (0.07) | 6 (0.10) | 1.43 (0.44 – 4.66) | 0.555 | 204.9 | 1.30 (0.39 – 4.28) | 0.669 | 198.8 |
| Overdominant |  |  |  |  |  |  |  |  |
| TT-CC | 36 (0.42) | 23 (0.37) | 1.22 (0.62 – 2.39) | 0.558 | 204.9 | 1.45 (0.72 – 2.92) | 0.298 | 197.9 |
| CT | 50 (0.58) | 39 (0.63) |  |  |  |  |  |  |
| Log-Additive  0,1,2 | 86 (0.58) | 62 (0.42) | 1.35 (0.77 – 2.36) | 0.296 | 204.2 | 1.45 (0.81 – 2.60) | 0.205 | 197.4 |
| ***IL18* rs187238** | | | | | | | | |
| Codominant |  |  |  |  |  |  |  |  |
| GG | 32 (0.37) | 31 (0.50) |  |  |  |  |  |  |
| GC | 46 (0.53) | 25 (0.40) | 0.56 (0.28 – 1.12) | 0.259 | 204.6 | 0.64 (0.31 – 1.31) | 0.469 | 199.5 |
| CC | 8 (0.09) | 6 (0.10) | 0.77 (0.24 – 2.49) |  |  | 0.80 (0.24 – 2.68) |  |  |
| Dominant |  |  |  |  |  |  |  |  |
| GG | 32 (0.37) | 31 (0.50) | 0.59 (0.31 – 1.15) | 0.120 | 202.9 | 0.67 (0.34 – 1.31) | 0.240 | 197.6 |
| GC-CC | 54 (0.63) | 31 (0.50) |  |  |  |  |  |  |
| Recessive |  |  |  |  |  |  |  |  |
| GG-GC | 78 (0.91) | 56 (0.90) |  |  |  |  |  |  |
| CC | 8 (0.09) | 6 (0.10) | 1.04 (0.34 – 3.18) | 0.938 | 205.3 | 1.00 (0.32 – 3.18) | 0.996 | 199.0 |
| Overdominant |  |  |  |  |  |  |  |  |
| GG-CC | 40 (0.46) | 37 (0.60) | 0.59 (0.30 – 1.14) | 0.112 | 202.7 | 0.66 (0.34 – 1.32) | 0.239 | 197.6 |
| GC | 46 (0.54) | 25 (0.40) |  |  |  |  |  |  |
| Log-Additive  0,1,2 | 86 (0.58) | 62 (0.42) | 0.74 (0.44 – 1.24) | 0.242 | 203.9 | 0.79 (0.46 – 1.33) | 0.366 | 198.2 |
| ***P2RX7* rs2230911** | | | | | | | | |
| Codominant |  |  |  |  |  |  |  |  |
| CC | 50 (0.58) | 40 (0.64) |  |  |  |  |  |  |
| CG | 34 (0.40) | 22 (0.36) | 0.81 (0.41 – 1.59) | 0.525 | 204.7 | 0.92 (0.45 – 1.87) | 0.535 | 199.8 |
| GG | 2 (0.02) | 0 | - | - | - | - | - | - |
| Dominant |  |  |  |  |  |  |  |  |
| CC | 50 (0.58) | 40 (0.64) | 0.76 (0.39 – 1.50) | 0.432 | 204.6 | 0.89 (0.44 – 1.81) | 0.740 | 198.9 |
| CG-GG | 36 (0.42) | 22 (0.36) |  |  |  |  |  |  |
| Recessive |  |  |  |  |  |  |  |  |
| CC-CG | 84 (0.98) | 62 (100) |  |  |  |  |  |  |
| GG | 2 (0.02) | 0 | - | - | - | - | - | - |
| Overdominant |  |  |  |  |  |  |  |  |
| CC-GG | 52 (0.60) | 40 (0.65) | 0.84 (0.43 – 1.65) | 0.615 | 205.0 | 0.94 (0.46 – 1.92) | 0.865 | 199.0 |
| CG | 34 (0.40) | 22 (0.35) |  |  |  |  |  |  |
| Log-Additive  0,1,2 | 86 (0.58) | 62 (0.42) | 0.72 (0.38 – 1.37) | 0.525 | 204.2 | 0.85 (0.42 – 1.68) | 0.633 | 198.8 |
| ***P2RX7* rs3751143** | | | | | | | | |
| Codominant |  |  |  |  |  |  |  |  |
| AA | 52 (0.60) | 32 (0.52) |  |  |  |  |  |  |
| AC | 33 (0.38) | 27 (0.43) | 1.33 (0.68 – 2.61) | 0.279 | 204.7 | 1.28 (0.64 – 2.56) | 0.363 | 199.0 |
| CC | 1 (0.02) | 3 (0.05) | 4.87 (0.49 – 48.90) |  |  | 4.27 (0.42 – 43.61) |  |  |
| Dominant |  |  |  |  |  |  |  |  |
| AA | 52 (0.60) | 32 (0.52) | 1.43 (0.74 – 2.77) | 0.283 | 204.1 | 1.38 (0.70 – 2.71) | 0.353 | 198.2 |
| AC-CC | 34 (0.40) | 30 (0.48) |  |  |  |  |  |  |
| Recessive |  |  |  |  |  |  |  |  |
| AA-AC | 85 (0.99) | 59 (0.95) |  |  |  |  |  |  |
| CC | 1 (0.01) | 3 (0.05) | 4.32 (0.44 – 42.57) | 0.172 | 203.4 | 3.84 (0.38 – 38.49) | 0.216 | 197.5 |
| Overdominant |  |  |  |  |  |  |  |  |
| AA-CC | 53 (0.62) | 35 (0.56) | 1.24 (0.64 – 2.41) | 0.527 | 204.9 | 1.20 (0.61 – 2.37) | 0.601 | 198.7 |
| AC | 33 (0.48) | 27 (0.44) |  |  |  |  |  |  |
| log-Addtive  0,1,2 | 86 (0.58) | 62 (0.42) | 1.51 (0.83 – 2.74) | 0.172 | 203.4 | 1.45 (0.79 – 2.67) | 0.230 | 197.6 |
| ***NLRP1* rs35865013** | | | | | | | | |
| Codominant |  |  |  |  |  |  |  |  |
| AA | 13 (0.15) | 14 (0.23) |  |  |  |  |  |  |
| AG | 73 (0.85) | 48 (0.77) | 0.61 (0.26 – 1.41) | 0.248 | 203.9 | 0.58 (0.24 – 1.41) | 0.226 | 197.6 |
| GG | 0 | 0 | - | - | - |  |  |  |
| log-Additive  0,1,2 | 86 (0.58) | 62 (0.42) | - | - | - | 0.58 (0.24 – 1.41) | - | 197.6 |
| ***NLRP3* rs10754558** | | | | | | | | |
| Codominant |  |  |  |  |  |  |  |  |
| CC | 50 (0.58) | 36 (0.58) |  |  |  |  |  |  |
| CG | 32 (0.37) | 20 (0.32) | 0.87 (0.43 – 1.76) | 0.454 | 205.7 | 0.85 (0.41 – 1.76) | 0.466 | 199.5 |
| GG | 4 (0.05) | 6 (0.10) | 2.08 (0.55 – 7.92) |  |  | 2.07 (0.52 – 8.18) |  |  |
| Dominant |  |  |  |  |  |  |  |  |
| CC | 50 (0.58) | 36 (0.58) | 1.00 (0.52 – 1.94) | 0.992 | 205.3 | 0.99 (0.50 – 1.95) | 0.974 | 199.0 |
| CG-GG | 36 (0.42) | 26 (0.42) |  |  |  |  |  |  |
| Recessive |  |  |  |  |  |  |  |  |
| CC-CG | 82 (0.95) | 56 (0.90) |  |  |  |  |  |  |
| GG | 4 (0.05) | 6 (0.10) | 2.20 (0.59 – 8.14) | 0.233 | 203.8 | 2.20 (0.57 – 8.46) | 0.246 | 197.7 |
| Overdominant |  |  |  |  |  |  |  |  |
| CC-GG | 54 (0.63) | 42 (0.68) | 0.80 (0.40 – 1.60) | 0.532 | 204.9 | 0.79 (0.39 – 1.61) | 0.515 | 198.6 |
| CG | 32 (0.47) | 20 (0.32) |  |  |  |  |  |  |
| log-Additive  0,1,2 | 86 (0.58) | 62 (0.42) | 1.14 (0.68 – 1.93) | 0.622 | 205.0 | 1.13 (0.66 – 1.94) | 0.655 | 198.8 |
| ***NLRP3* rs10805502** | | | | | | | | |
| Codominant |  |  |  |  |  |  |  |  |
| CC | 35 (0.41) | 28 (0.45) |  |  |  |  |  |  |
| CT | 45 (0.52) | 26 (0.42) | 0.72 (0.36 – 1.44) | 0.316 | 205.0 | 0.84 (0.41 – 1.71) | 0.487 | 199.6 |
| TT | 6 (0.07) | 8 (0.13) | 1.67 (0.52 – 5.37) |  |  | 1.72 (0.52 – 5.70) |  |  |
| Dominant |  |  |  |  |  |  |  |  |
| CC | 35 (0.41) | 28 (0.45) | 0.83 (0.43 – 1.61) | 0.588 | 205.0 | 0.95 (0.48 – 1.88) | 0.890 | 199.0 |
| CT-TT | 51 (0.59) | 34 (0.55) |  |  |  |  |  |  |
| Recessive |  |  |  |  |  |  |  |  |
| CC-CT | 80 (0.93) | 54 (0.87) |  |  |  |  |  |  |
| TT | 6 (0.07) | 8 (0.13) | 1.98 (0.65 – 6.01) | 0.227 | 203.8 | 1.88 (0.60 – 5.89) | 0.272 | 197.8 |
| Overdominant |  |  |  |  |  |  |  |  |
| CC-TT | 41 (0.48) | 36 (0.58) | 0.66 (0.34 – 1.27) | 0.211 | 203.7 | 0.76 (0.39 – 1.50) | 0.426 | 198.4 |
| CT | 45 (0.52) | 26 (0.42) |  |  |  |  |  |  |
| log-Additive  0,1,2 | 86 (0.58) | 62 (0.42) | 1.04 (0.62 – 1.72) | 0.891 | 205.2 | 1.11 (0.66 – 1.87) | 0.688 | 198.9 |

Legend: Adjusted for sex and age (p value_ad_j, OR_adj_); OR: Odds Ratio; p value: < 0.05; 95% confidence interval; AIC: Akaike information criterion value.

**Supplementary Table S3.** Allele frequencies of NLRP1, NLRP3, P2RX7, IL1B and IL18 polymorphisms in patients with acute lymphoblastic leukemia and healthy controls.

|  | Controls vs. ALL | | | | Infectious comorbidities | | | | Relapse | | | | Death | | | |
| --- | --- | --- | --- | --- | --- | --- | --- | --- | --- | --- | --- | --- | --- | --- | --- | --- |
| Alleles | **Controls**  **(n= 192)** | **ALL cases**  **(n=158)** | **OR**  **(IC 95%)** | ***p* value** | **No**  **(n= 86)** | **Yes**  **(n= 62)** | **OR**  **(IC 95%)** | ***p* value** | **No**  **(n=53)** | **Yes**  **(n=105)** | **OR**  **(IC 95%)** | ***p* value** | **No**  **(n= 92)** | **Yes**  **(n= 66)** | **OR**  **(IC 95%)** | ***p* value** |
| *IL1B rs16944 C/T* | | | | | | | | | | | | | | | | |
| C | 165 (42%) | 123 (38%) | 0.84  (0.62 – 1.14) | 0.279 | 63 (37%) | 51 (41%) | 1.20  (0.75 – 1.92) | 0.468 | 43 (40%) | 80 (38%) | 0.90  (0.55 – 1.45) | 0.670 | 69 (37%) | 54 (40%) | 1.15  (0.73 – 1.82) | 0.539 |
| T | 219 (58%) | 193 (62%) |  |  | 109 (63%) | 73 (59%) |  |  | 63 (80%) | 130 (62%) |  |  | 115 (63%) | 78 (60%) |  |  |
| *IL18 rs187238 G/C* | | | | | | | | | | | | | | | | |
| G | 254 (66%) | 211 (66%) | 1.02 (0.75 – 1.41) | 0.861 | 110 (64%) | 87 (70%) | 1.32  (0.80 – 2.17) | 0.317 | 66 (62%) | 145 (69%) | 1.35  (0.82 – 2.20) | 0.226 | 125 (68%) | 86 (65%) | 0.88  (0.54 – 1.41) | 0.604 |
| C | 130 (34%) | 105 (44%) |  |  | 62 (36%) | 37 (30%) |  |  | 40 (38%) | 65 (31%) |  |  | 59 (32%) | 46 (35%) |  |  |
| *P2RX7 rs2230911 C/G* | | | | | | | | | | | | | | | | |
| C | 313 (81%) | 253 (80%) | 0.91 (0.62 – 1.32) | 0.628 | 135 (78%) | 102 (82%) | 1.27  (0.72 – 2.25) | 0.463 | 86 (81%) | 167 (80%) | 0.90  (0.50 – 1.63) | 0.735 | 144 (78%) | 109 (82%) | 1.31  (0.74 – 2.32) | 0.343 |
| G | 71 (19%) | 63 (20%) |  |  | 37 (22%) | 22 (18%) |  |  | 20 (19%) | 43 (20%) |  |  | 40 (22%) | 23 (18%) |  |  |
| *P2RX7 rs3751143 A/C* | | | | | | | | | | | | | | | | |
| A | 313 (81%) | 244 (77%) | 0.76 (0.53 – 1.11) | 0.160 | 135 (78%) | 91 (73%) | 0.75  (0.44 – 1.29) | 0.333 | 83 (78%) | 161 (77%) | 0.91  (0.51 – 1.59) | 0.743 | 146 (79%) | 98 (74%) | 0.75  (0.44 – 1.27) | 0.285 |
| C | 71 (19%) | 72 (33%) |  |  | 37 (22%) | 33 (27%) |  |  | 23 (22%) | 49 (33%) |  |  | 38 (21%) | 34 (26%) |  |  |
| *NLRP1 rs35865013 A/G* | | | | | | | | | | | | | | | | |
| A | 217 (56%) | 187 (59%) | 1.11 (0.82 – 1.50) | 0.477 | 99 (58%) | 76 (61%) | 1.16  (0.73 – 1.86) | 0.550 | 67 (63%) | 120 (57%) | 0.77  (0.48 – 1.25) | 0.300 | 111 (60%) | 76 (57%) | 0.89  (0.56 – 1.40) | 0.623 |
| G | 167 (44%) | 129 (41%) |  |  | 73 (42%) | 48 (39%) |  |  | 39 (37%) | 90 (86%) |  |  | 73 (40%) | 56 (43%) |  |  |
| *NLRP1 rs12150220 A/T* | | | | | | | | | | | | | | | | |
| A | 250 (65%) | 210 (66%) | 1.06 (0.77 – 1.45) | 0.707 | 109 (63%) | 84 (68%) | 1.21  (0.74 – 1.95) | 0.460 | 66 (62%) | 144 (68%) | 1.32  (0.81 – 2.15) | 0.262 | 123 (71%) | 87 (61%) | 0.80  (0.49 – 1.30) | 0.372 |
| T | 134 (35%) | 106 (44%) |  |  | 63 (37%) | 40 (32%) |  |  | 40 (38%) | 66 (32%) |  |  | 51 (29%) | 45 (39%) |  |  |
| *NLRP3 rs10754558 C/G* | | | | | | | | | | | | | | | | |
| C | 297 (77%) | 240 (75%) | 0.92 (0.65 – 1.31) | 0.664 | 134 (78%) | 92 (74%) | 0.81  (0.48 – 1.40) | 0.489 | 77 (72%) | 163 (78%) | 1.30  (0.76 – 2.23) | 0.328 | 135 (73%) | 105 (80%) | 1.41  (0.82 – 2.40) | 0.205 |
| G | 87 (33%) | 76 (25%) |  |  | 38 (22%) | 32 (26%) |  |  | 29 (28%) | 47 (22%) |  |  | 49 (27%) | 27 (20%) |  |  |
| *NLRP3 rs10805502 C/T* | | | | | | | | | | | | | | | | |
| C | 241 (63%) | 208 (65%) | 1.14 (0.83 – 1.56) | 0.400 | 115 (67%) | 82 (66%) | 0.96  (0.59 – 1.55) | 0.901 | 64 (60%) | 144 (68%) | 1.43  (0.88 – 2.32) | 0.147 | 119 (65%) | 89 (67%) | 1.13  (0.70 – 1.81) | 0.611 |
| T | 143 (37%) | 108 (35%) |  |  | 57 (33%) | 42 (34%) |  |  | 42 (40%) | 66 (32%) |  |  | 65 (35%) | 43 (33%) |  |  |

Legend: OR: Odds Ratio; p value: < 0.05; 95% confidence interval.

**Supplementary Table S4.** The probes used in the study.

| Gene | Identification variant | Location on chromosome | Probe sequence 5’-VIC/FAM-3’ |
| --- | --- | --- | --- |
| *NLRP1* | rs12150220 | Chr.17:  5582047 | GGAGCTTGGAAGAGCTTGGTAGAGG**[A/T]**GTGAGGCAGAGATTTCTGGGGGGAA |
|  | rs35865013 | Chr17:  5512596 | GGTAAAGGAGATGGGTGGGTGGGGC**[A/G]**TGAAGGACCCGAAAGACAAGGCCAG |
| *NLRP3* | rs10754558 | Chr1:  247448734 | GACAATGACAGCATCGGGTGTTGTT**[C/G]**TCATCACAGCGCCTCAGTTAGAGGA |
|  | rs10805502 | Chr1:  247448993 | ATTTATTTATTTAAATTTTTTGTAA**[C/T]**AGTTTTGTTTTCTAATAAGAAAAAT |
| *P2X7* | rs3751143 | Chr12:  121184501 | CCTGAGAGCCACAGGTGCCTGGAGG**[A/C]**GCTGTGCTGCCGGAAAAAGCCGGGG |
|  | rs2230911 | Chr12:  121177328 | GTGTTCATCGACTTCCTCATCGACA**[C/G]**TTACTCCAGTAACTGCTGTCGCTCC |
